# Supplementary material for: A FN-MdV pathway and its role in cerebellar multimodular control of sensorimotor behavior
Source: Nat Commun. 2020 Nov 27;11:6050. doi: 10.1038/s41467-020-19960-x (PMC7695696; doi:10.1038/s41467-020-19960-x)
Supplement: Supplementary file 1 — Supplementary information [file 41467_2020_19960_MOESM1_ESM.pdf]

Supplementary Information

A FN-MdV Pathway and Its Role in Cerebellar Multimodular Control of Sensorimotor Behavior

Wang *et al*

Supplementary Figures 1-11

Supplementary Table 1

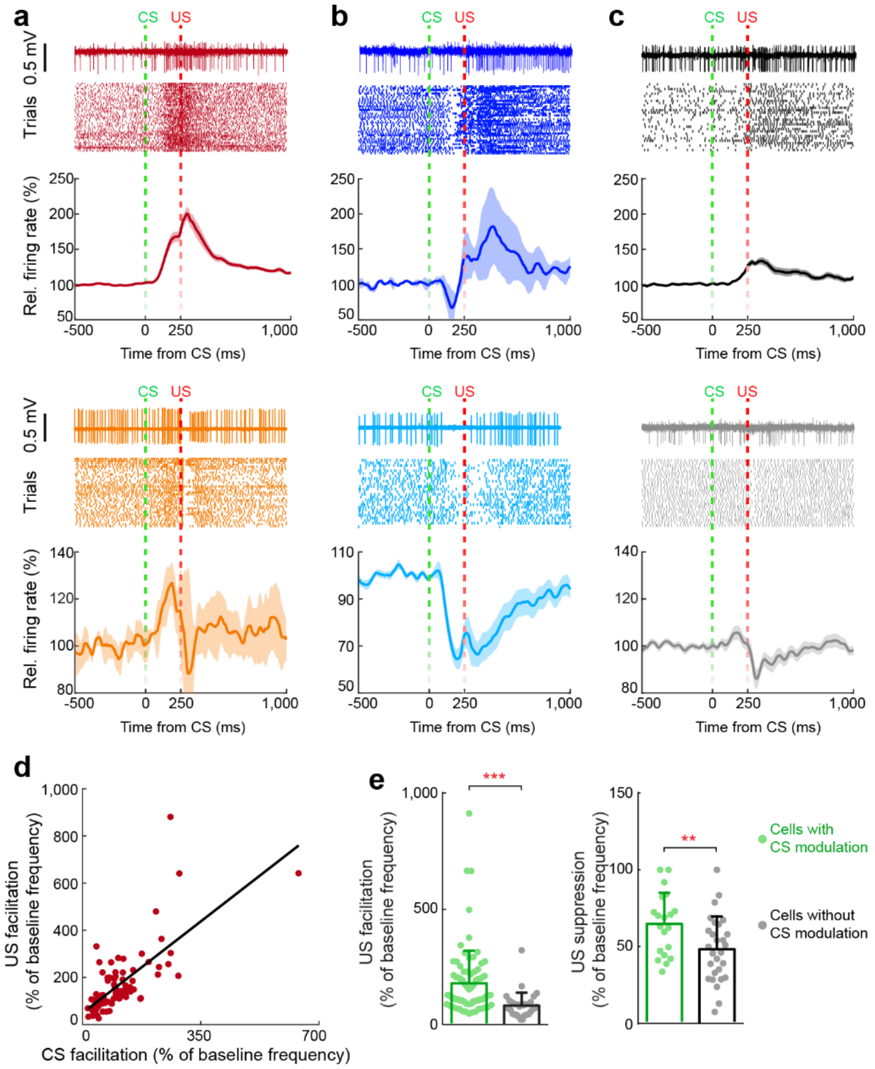

Supplementary Figure 1. US-related modulation in FN neurons. Data related to Fig. 1

a, US-related facilitation (top red panel, n = 80 neurons) and suppression (bottom orange panel, n = 6 neurons) in FN neurons exhibiting CS-related facilitation. In each panel, top row: example recording of a single trial from a representative cell; middle row: raster plot of spike events of the same cell; bottom row: group summary of spike modulation of all neurons with US-related facilitation (mean ± s.e.m.). **b**, **c**, Same as **a**, but for the US-related modulations in FN neurons exhibiting CS-related suppression (**b**; upper dark blue: n = 3, lower light blue: n = 13) and no significant modulation (**c**; upper black: n = 31, lower gray: n = 29), traces are plotted as mean ± s.e.m.. **d**, Correlation between the CS- and US-related facilitation for neurons in the (**a**) red group (linear regression model, $P = 1.4\times10^{-13}$, $r^2 = 0.5$). **e**, Summary of the US-related modulation (mean ± SD, left: facilitation, n = 113; right: suppression, n = 49) in the FN neurons with and without CS-related modulation (***$P = 1.36\times10^{13}$, **P = 0.00041, two-sample t-test (two-sided)).

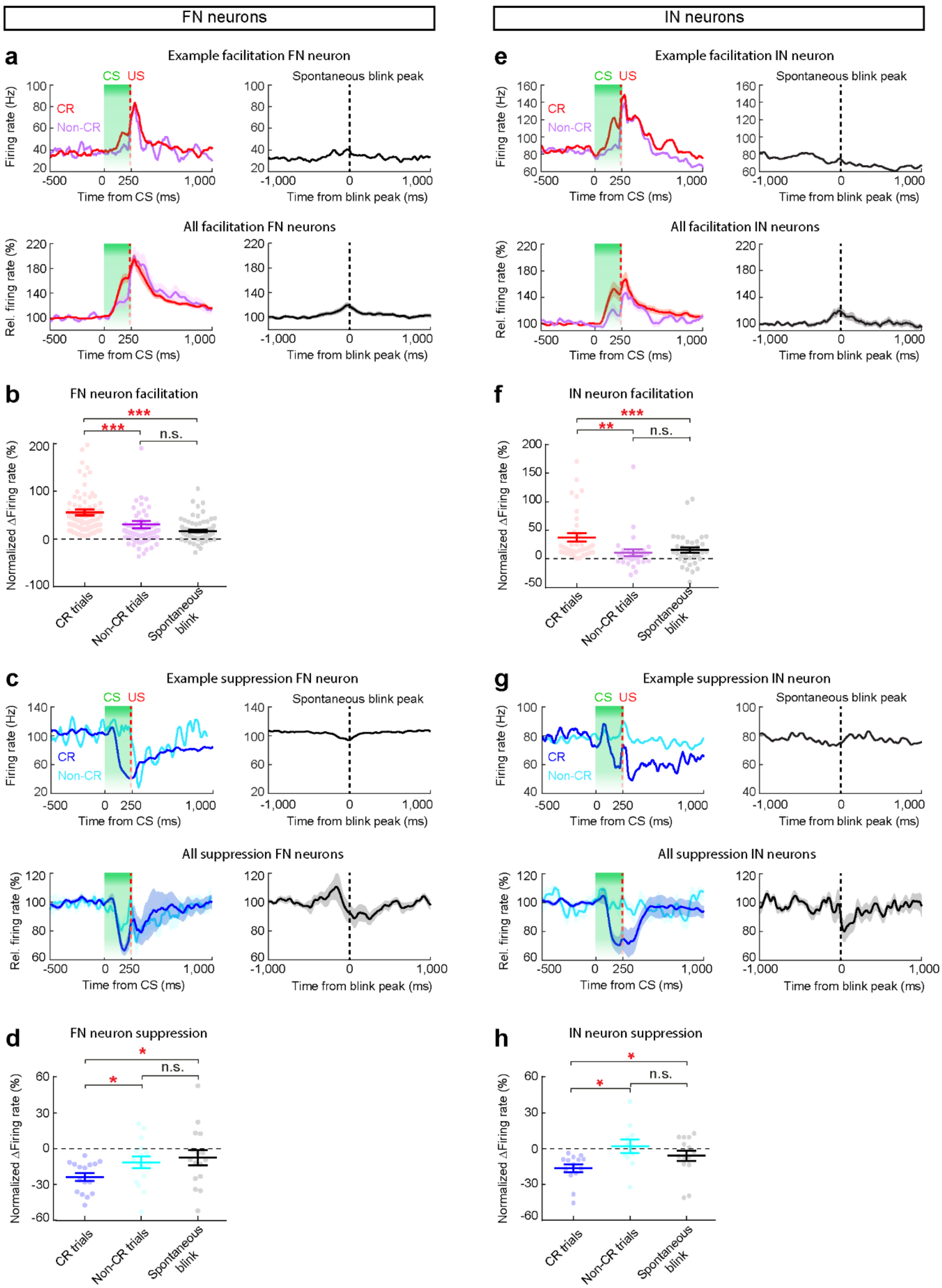

FN neurons
IN neurons
a
Example facilitation FN neuron
CS US
CR
Non-CR
Spontaneous blink peak
Firing rate (Hz)
Time from CS (ms)
Time from blink peak (ms)
All facilitation FN neurons
Rel. firing rate (%)
b
FN neuron facilitation
***
***
n.s.
Normalized ΔFiring rate (%)
CR trials
Non-CR trials
Spontaneous blink
c
Example suppression FN neuron
All suppression FN neurons
d
FN neuron suppression
*
*
n.s.
e
Example facilitation IN neuron
All facilitation IN neurons
f
IN neuron facilitation
***
**
n.s.
g
Example suppression IN neuron
All suppression IN neurons
h
IN neuron suppression
*
*
n.s.

Supplementary Figure 2. FN and IN neuron activity during non-CR trials and spontaneous blink. Data related to Fig. 1

a, FN neuron activity in CR trials (red), non-CR trials (magenta) and spontaneous blink (black). Upper: spike rates of an example neuron showing significant facilitation in CR trials, but not in non-CR trials or spontaneous blink; Lower: population average of all facilitation neurons in CR trials, non-CR trials and spontaneous blink (n = 86, mean ± s.e.m.). **b**, Comparison of the neuron activity in CR trials, non-CR trials (during 50-250 ms after CS onset) and spontaneous blink (within 100 ms before and after spontaneous blink peak), confirming that FN neuron facilitation is CR-related (mean ± s.e.m., n = 86, paired two-sided t-test, *$P < 0.05$, **$P < 0.01$, ***$P < 0.001$). **c,d**, Same as **a,b**, but for FN neuron suppression in CR trials (blue), non-CR trials (cyan) and spontaneous blink (black). Upper: an example neuron; Lower: all suppression neurons (n = 16, mean ± s.e.m.). **e-h**, Same as **a-d**, but for IN neurons. Dataset was also shown in our previous work (first dataset, M. Ten Brinke, *et al.*, 2017, eLife), n = 30 for facilitation IN neurons (**f**, mean ± s.e.m., paired two-sided t-test, **$P < 0.01$, ***$P < 0.001$), and n = 16 for suppression IN neurons (**h**, mean ± s.e.m., paired two-sided t-test, *$P < 0.05$), traces are plotted as mean ± s.e.m. (lower panels of **e** and **g**). See the exact P values for each comparison in the Source Data file.

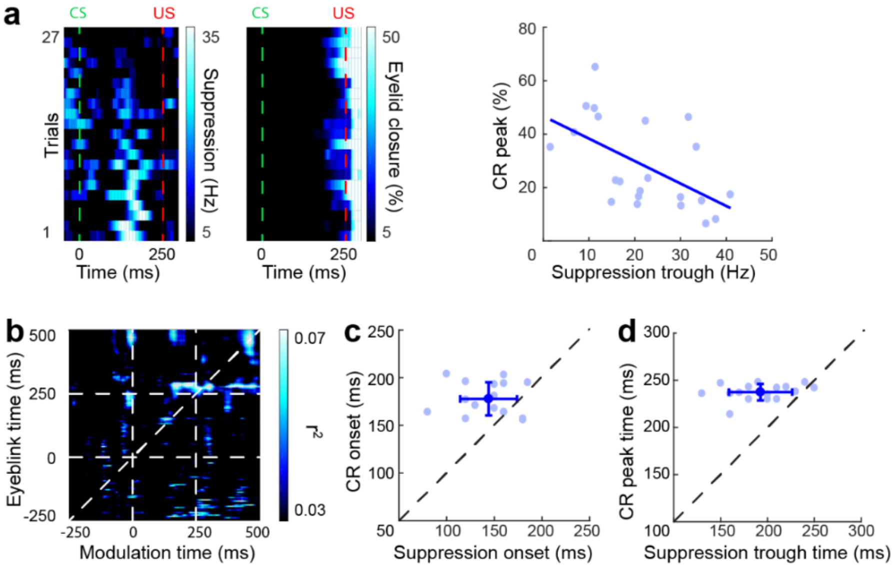

Supplementary Figure 3. Trial-by-trial correlation between FN suppression and CR amplitudes, Data related to Fig. 1

a, Example FN neuron with a trial-by-trial correlation between suppression and CR peak amplitudes. Left: Each row on the left heatmap represents a single trial of recording, and on the right heatmap represents the corresponding CR amplitudes of the same trials. All trials are ordered based on their suppression amplitudes. Dashed lines indicate CS and US onsets. Right: each dot represents a single trial; a negative correlation between neuron suppression and CR amplitudes of this cell (linear regression model, P = 0.0066). **b**, Average correlation matrix of 16 suppression cells. Each epoch indicates the mean r^2 value of trial-by-trial correlation between the FN neuron activity and eyelid closure at a given time point throughout the task. All epochs are minimally-correlated (dark pixels) within the CS-US interval. CS and US onsets are denoted with dashed lines in both dimensions. **c**, Relationship between the onset timings of neuronal modulation and the CR for all suppression cells, note the neuronal modulation occurred earlier than CR onset (mean ± SD, n = 16, paired two-sided t-test, P = 0.0019). **d**, Same as **c**, but for the comparison of the peak timing of neuronal modulation and the trough timing of CR for all suppression cells (n = 16, paired two-sided t-test, $P = 5.35\times10^{-5}$).

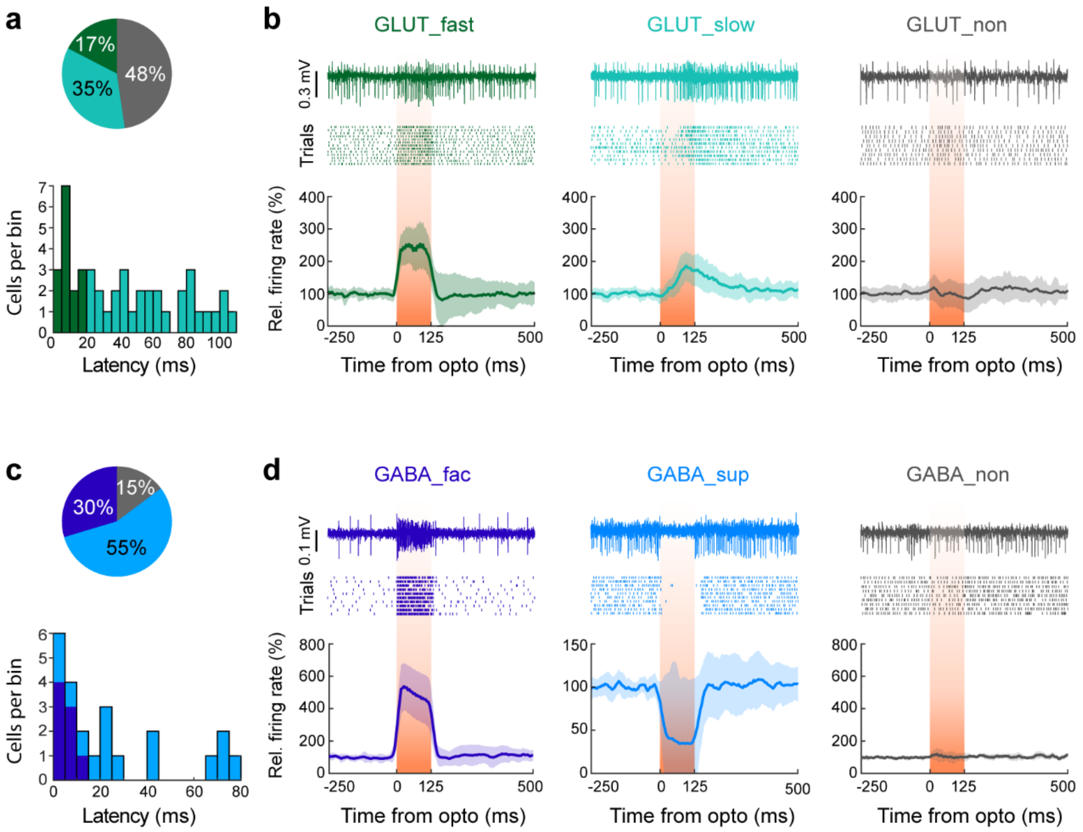

Supplementary Figure 4. FN neuron responses to optogenetic activation in VGluT2-Cre and Gad2-Cre mice. Data related to Fig. 2

a, Quantification of the facilitation latency in response to optogenetic activation of ChrimsonR in the FN neurons, in which ChrimsonR was expressed specifically in the VGluT2-cre+ cells. Summary of the fraction (upper) and the numbers of FN neurons (lower) in the VGluT2-Cre mice showing short-latency facilitation (onset < 20 ms, dark green), long-latency facilitation (onset ≥ 20 ms, light green) and no modulation (gray). **b**, Different neuron responses to the optogenetic activation in the VGluT2-Cre mice. From left to right: neuron responses to the optic light (orange shading) with short-latency facilitation (GLUT_fast, n = 15), long-latency facilitation (GLUT_slow, n = 30) and no modulation (GLUT_non, n = 41). Top row: example traces of single optogenetic stimulation trial; middle row: raster plot of spike events for each representative cell; bottom row: average neural activity of each group (mean ± s.e.m.). **c**, Same as **a**, but for cells with short-latency facilitation (onset < 20 ms, dark blue) and suppression (light blue) in the Gad2-Cre mice. **d**, Same as **b**, but for the recordings from Gad2-Cre mice. From left to right: neuron responses to optogenetics with short-latency facilitation (GABA_fac, n = 8), suppression (GABA_sup, n = 15) and no modulation (GABA_non, n = 4), traces are plotted as mean ± s.e.m..

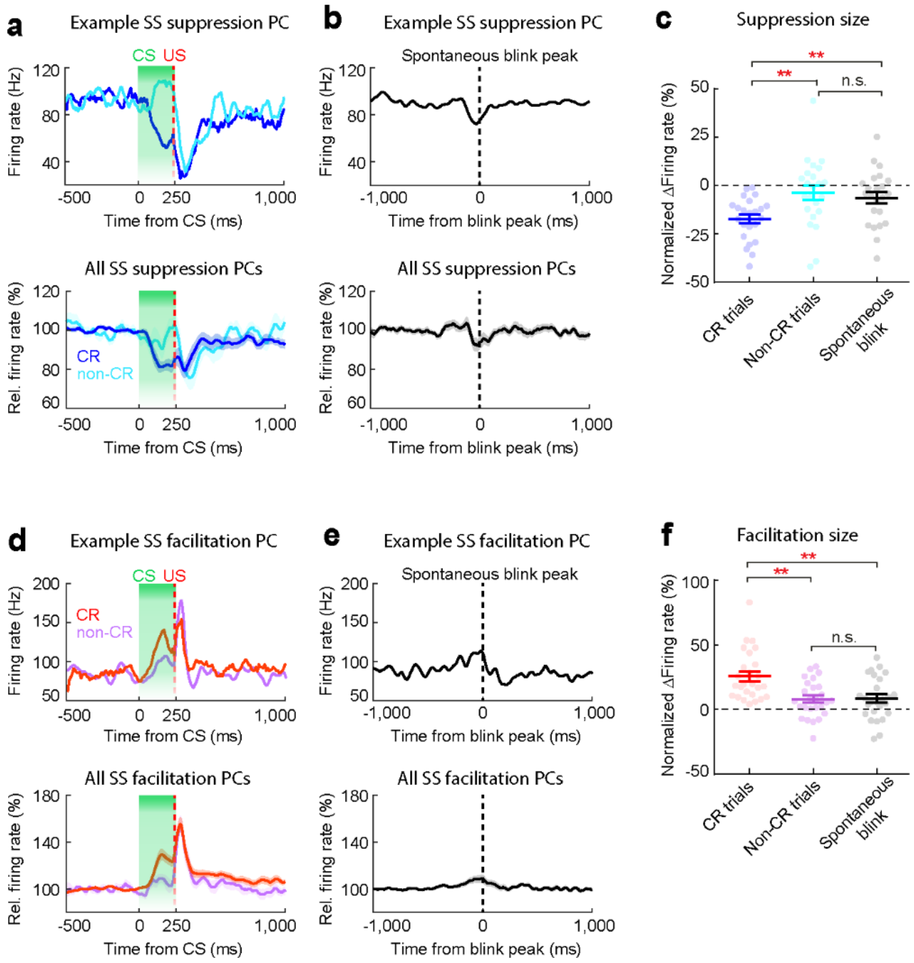

Supplementary Figure 5. Simple spike activity of vermal PCs in CR trials, non-CR trials and spontaneous blink. Data related to Fig. 3

a, Vermal PC show prominent simple spike suppression (SS suppression) in CR trials but not in non-CR trials. Upper: an example PC showing significant SS suppression in CR trials but not in non-CR trials; Lower: population activity of all SS suppression PCs (n = 23, mean ± s.e.m.). **b**, SS modulation of PCs during spontaneous blink. Upper: the same example PC as in **a**, showing no significant change of firing rate during spontaneous blink; Lower, population activity of all SS suppression PCs (n = 23, mean ± s.e.m.). Neuron activity is aligned to the peak of spontaneous blinking events. **c**, Summary of the SS suppression during CR trials, non-CR trials (during 50-250 ms after CS onset) and spontaneous blink (within 100 ms before and after spontaneous blink peak). Mean ± s.e.m., n = 23, paired two-sided t-test, $^{**}P < 0.01$. **d-f**, Same as **a-c**, but for vermal PCs with simple spike facilitation (SS facilitation). Upper: an example PC; Lower: all PCs with SS facilitation (n = 26, mean ± s.e.m., paired two-sided t-test, $^{**}P < 0.01$). See the exact P values for each comparison in the Source Data file.

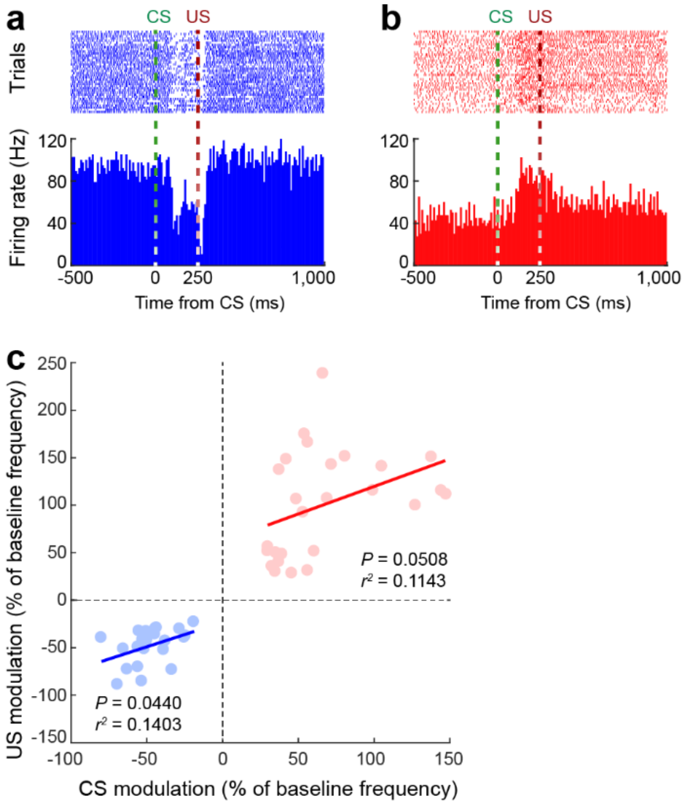

Supplementary Figure 6. US-related simple spike modulation in vermal PCs. Data related to Fig. 3

a, **b**, Raster plots of simple spikes (top) and corresponding PSTHs (bin size = 5 ms) of two example PCs showing suppression (**a**) and facilitation (**b**) in response to US. **c**, Correlation between CS- and US-related modulation (linear model regressions, n = 23 for blue cells, and n = 26 for red cells). Red dots denote cells with US-related facilitation, and blue dots denote cells with US-related suppression. See the exact P values for each regression in **c** and in the Source Data file.

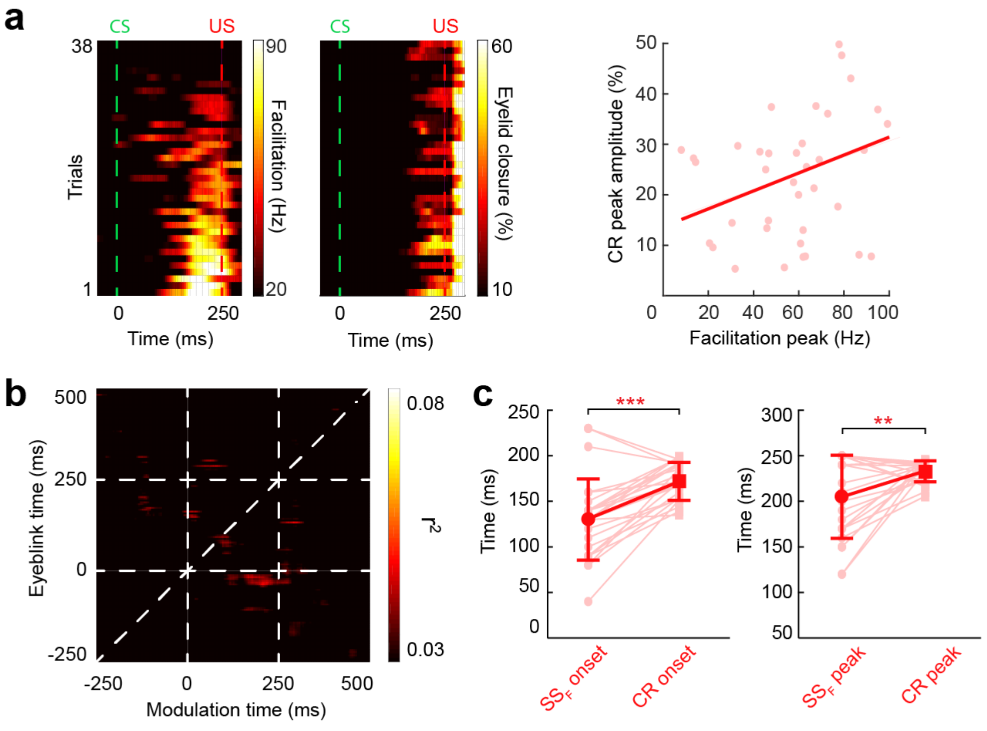

Supplementary Figure 7. Trial-by-trial correlation between simple spike facilitation and CR amplitudes, Data related to Fig. 3

a, Left panel, example PC with a positive correlation between simple spike activity and CR amplitudes. Each row on the left heatmap represents a single trial of recording, and on the right heatmap represents the corresponding CR amplitude of the same trial. Trials are ordered based on the facilitation peak amplitude. Dashed lines indicate the CS and US onsets. Right panel, positive correlation between the facilitation peak and the CR peak amplitude of the example cell (linear regression model, P = 0.031). **b**, Averaged correlation matrix of all 26 PCs with simple spike facilitation. Each epoch indicates the mean r^2 value of trial-by-trial correlation between the simple spike activity and the eyelid closure at a given time point throughout the task. No significant correlation is detected within the CS-US interval (dark pixels). CS (0 ms) and US (250 ms) onsets are denoted with dashed lines in both dimensions. **c**, Comparison between the timings of simple spike facilitation and the timings of CR. Simple spike facilitation precedes behavior in both the onset (left, mean ± SD, n = 26, paired two-sided t-test, ***$P = 4.04\times10^{-6}$) and peak timing (right, n = 26, paired two-sided t-test, **P = 0.0055).

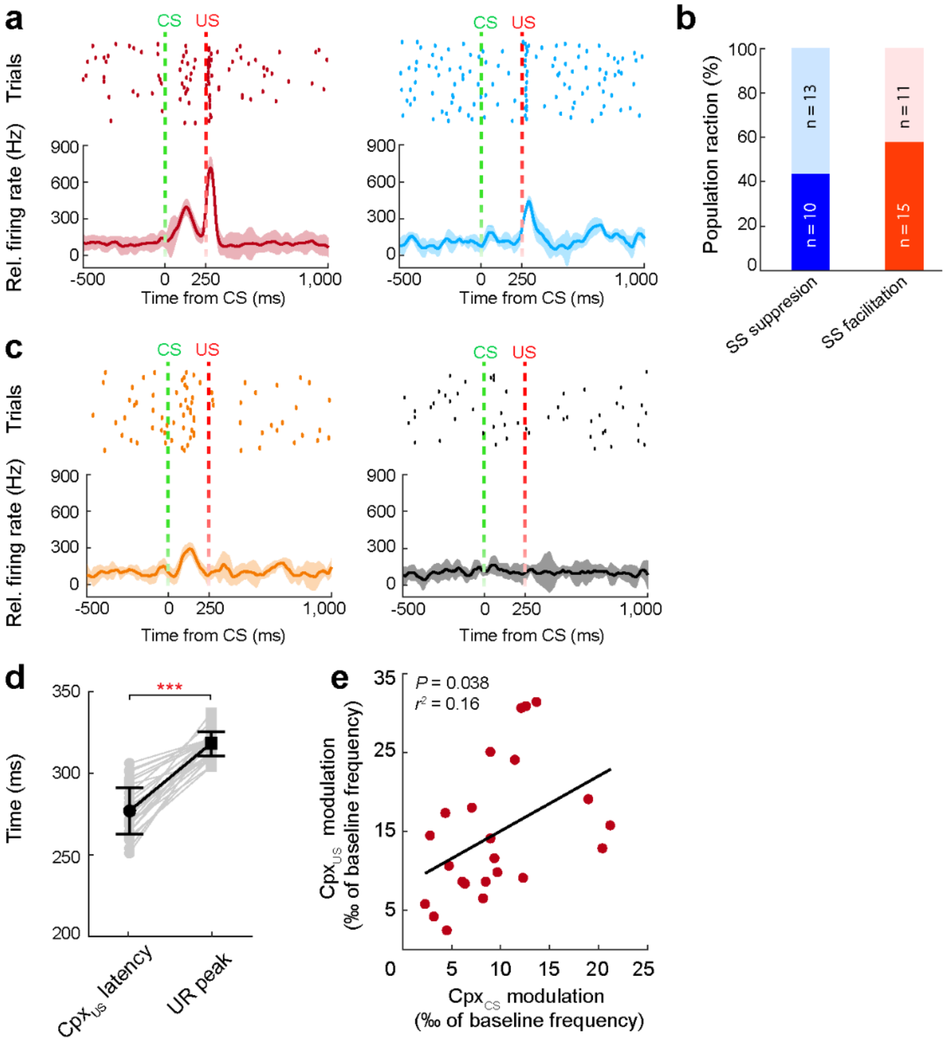

Supplementary Figure 8. US-related complex spike modulation in vermal PCs. Data related to Fig. 4

a, Short-latency US-related complex spikes (Cpx_{US}) in PCs exhibiting CS-related complex spikes (Cpx_{CS}, red traces, *n* = 23 neurons) and no Cpx_{CS} (blue traces, *n* = 6 neurons). Top: raster plots of complex spike events of two example cells; bottom: group average complex spike activity for each type of cells (mean ± s.e.m.). **b**, Population fraction of SS suppression PCs (*n* = 23) and SS facilitation PCs (*n* = 26) exhibiting short-latency Cpx_{US} (dark blue and red bars). **c**, Same as **a**, but for PCs without Cpx_{US} (*n* = 6 for orange trace and *n* = 8 for black trace, mean ± s.e.m.). **d**, Comparison of the Cpx_{US} latency and the UR peak time (paired two-sided *t*-test, ***$P = 9.69\times10^{-15}$, *n* = 29 neurons, mean ± SD). **e**, Correlation between CS- and US-related complex spike modulation in the PCs of (**a**, left; linear regression model, *P* = 0.038).

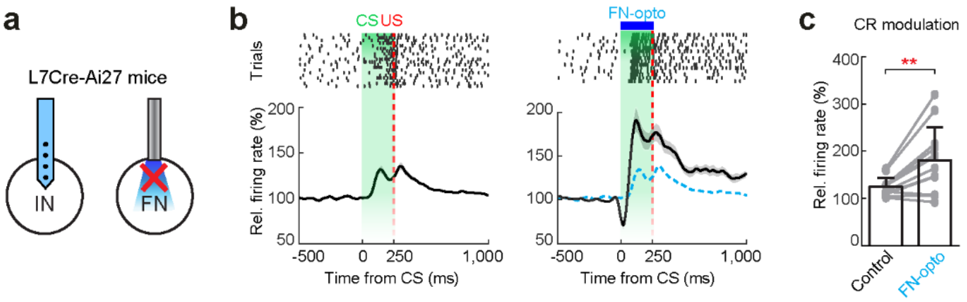

Supplementary Figure 9. Effects of Photo-inhibiting the FN on Modulation of the Ipsilateral IN. Data related to Fig. 5

a, Scheme showing IN neuron recording during DEC with photoinhibition of the ipsilateral FN in L7Cre-Ai27 mice. **b**, CS-related activity of IN neurons during control trials (left) and FN-inhibition trials (right). Upper: raster plots of an example IN neuron spike event. Lower: average activity pattern of all IN cells with CS-related facilitation (*n* = 12 neurons, mean ± s.e.m.). **c**, Comparison of CS-related facilitation of IN neurons in control and FN-inhibition trials. CS-related facilitation of IN neurons is enhanced in FN-inhibition trials compared to control trials (*n* = 12 neurons, mean ± SD, paired two-sided *t*-test, ***P* = 0.0028).

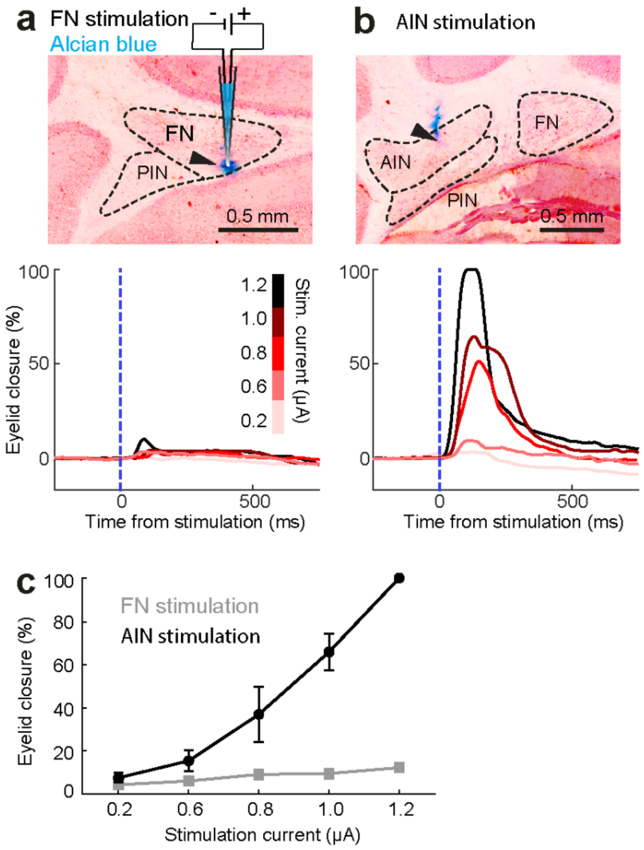

Supplementary Figure 10. Effects of electric stimulation in FN or AIN on eyelid movement. Data related to Fig. 5

a, **b**, Example histological sections showing the locations of electric stimulation in FN or AIN (upper panels) and the corresponding evoked eyelid closure with graded current intensities (lower panels) in naïve mice. Alcian blue was injected through the stimulation glass pipette to visualize the stimulation sites. Experiments were performed and replicated in 3 mice. **c**, Summary of eyelid closure amplitudes with different stimulation intensities in FN and AIN from 3 animals (mean ± s.e.m.). AIN: anterior interposed nucleus; PIN: posterior interposed nucleus; FN: fastigial nucleus.

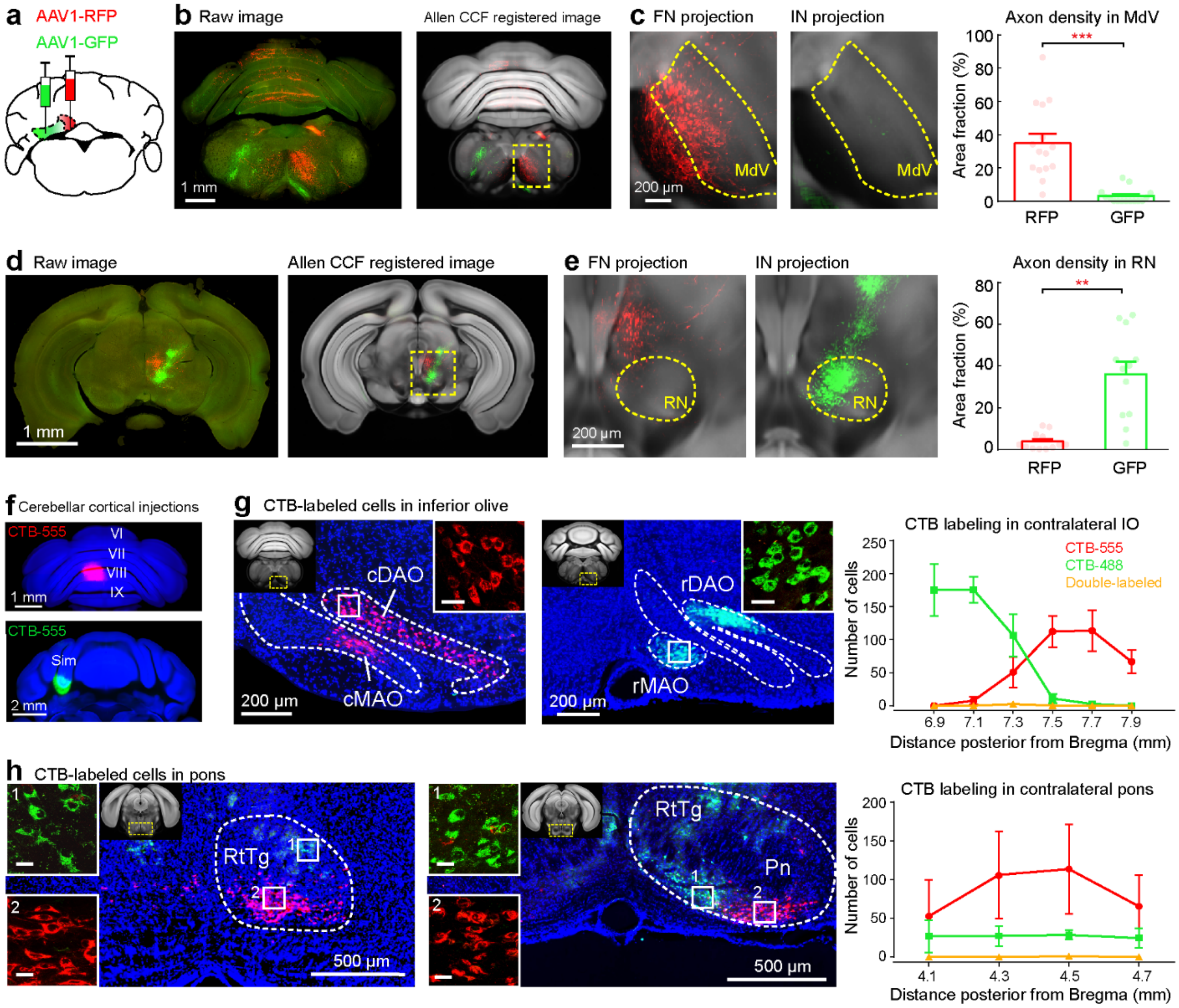

Supplementary Figure 11. Inputs and outputs of the simplex lobule-IN and the vermis-FN modules. Data related to Fig. 8

a, Tracing strategy to label FN (AAV1-RFP) and IN (AAV1-GFP) output projections. **b**, Coronal sections showing an example case of IN and FN projections at the level of caudal medulla. Raw image (left) is registered to the Allen Mouse Brain CCF (right, see Methods). Zoom-in image of the dashed-lined area containing MdV region is shown in **c**. Quantification of projection from FN and IN to MdV (**c**, *n* = 15 sections from 3 mice, mean ± s.e.m., Wilcoxon Signed Ranks Test (two-tailed), ****P* = 0.001). **d**, **e**, Same as **b**, **c**, but for projections in the RN (*n* = 12 sections from 3 mice, mean ± s.e.m., Wilcoxon Signed Ranks Test (two-tailed), ***P* = 0.002). IN: anterior interposed nucleus; RN: red nucleus; MdV: ventral medullary reticular nucleus. **f**, Coronal sections showing an example case with double fluorescent CTB injections in the vermis (red CTB, upper) and the simplex lobule (green CTB, lower). **g**, Coronal images and quantification of retrogradely-

labeled cells in the inferior olive of 3 mice (mean ± s.e.m.). Scale bars of inserted images are 20 μm. **h**, Same as **g**, but for quantification in in the Pn. Scale bars of inserted images are 20 μm. cMAO: caudal medial accessory olive; cDAO: caudal dorsal accessory olive; cMAO: caudal medial accessory olive; rMAO: rostral medial accessory olive; RtTg: reticulotegmental nucleus of the pons; Pn: pontine nucleus. Both tracing experiments (**a-e** and **f-h**) were performed and replicated in 3 mice.

Supplementary Table 1. Stereotaxic coordinates of nuclei*

Nuclei	AP (mm)	ML (mm)	Depth (mm)
Fastigial nucleus	2.7	0.8	2.4
Anterior interposed nucleus	2.3	2.0	2.3
Facial nucleus	1.9	1.5	4.6
Ventral medullary reticular nucleus	3.5	0.8	4.5

*The origin of coordinates is defined as the anterior tip of the interparietal bone. Viral/tracer injection volume was 20-50 nL. AP: anterior posterior, ML: medial lateral
