# Supplementary material for: A FN-MdV pathway and its role in cerebellar multimodular control of sensorimotor behavior
Source: Nat Commun. 2020 Nov 27;11:6050. doi: 10.1038/s41467-020-19960-x (PMC7695696; doi:10.1038/s41467-020-19960-x)
Supplement: Supplementary file 3 — Reporting Summary [file 41467_2020_19960_MOESM3_ESM.pdf]

nature research

Corresponding author(s): Zhenyu Gao

Last updated by author(s): Oct 26, 2020

Reporting Summary

Nature Research wishes to improve the reproducibility of the work that we publish. This form provides structure for consistency and transparency in reporting. For further information on Nature Research policies, see our Editorial Policies and the Editorial Policy Checklist.

Statistics

For all statistical analyses, confirm that the following items are present in the figure legend, table legend, main text, or Methods section.

n/a	Confirmed	
☐	☒	The exact sample size (n) for each experimental group/condition, given as a discrete number and unit of measurement
☐	☒	A statement on whether measurements were taken from distinct samples or whether the same sample was measured repeatedly
☐	☒	The statistical test(s) used AND whether they are one- or two-sided<br>*Only common tests should be described solely by name; describe more complex techniques in the Methods section.*
☒	☐	A description of all covariates tested
☐	☒	A description of any assumptions or corrections, such as tests of normality and adjustment for multiple comparisons
☐	☒	A full description of the statistical parameters including central tendency (e.g. means) or other basic estimates (e.g. regression coefficient) AND variation (e.g. standard deviation) or associated estimates of uncertainty (e.g. confidence intervals)
☐	☒	For null hypothesis testing, the test statistic (e.g. F, t, r) with confidence intervals, effect sizes, degrees of freedom and P value noted<br>*Give P values as exact values whenever suitable.*
☒	☐	For Bayesian analysis, information on the choice of priors and Markov chain Monte Carlo settings
☒	☐	For hierarchical and complex designs, identification of the appropriate level for tests and full reporting of outcomes
☒	☐	Estimates of effect sizes (e.g. Cohen's d, Pearson's r), indicating how they were calculated

Our web collection on statistics for biologists contains articles on many of the points above.

Software and code

Policy information about availability of computer code

Data collection	Electrophysiological recording and behavioral data were collected with Axon Acquisition Systems (1440A, Molecular Devices Corporation) and/or Intan Evaluation Systems (RHD2000, Intan Technology). Light microscopy images were collected with a Nanozoomer (2.0-RS, Hamamatsu). Fluorescence microscopy images were collected with a wide-field fluorescence scanner (Axio Imager 2, ZEISS) or a confocal microscope (LSM 700, ZEISS).
Data analysis	Electrophysiological and behavioral data were analyzed with Matlab 2014 and 2017. All statistics were performed with Matlab 2017, IBM SPSS Statistics 24 and GraphPad Prism 6. Brain images were processed by using ImageJ and Adobe Photoshop CC 2019. For the custom-made analytical Matlab codes, please see https://github.com/XiaoluOne/FN-eyeblink.

For manuscripts utilizing custom algorithms or software that are central to the research but not yet described in published literature, software must be made available to editors and reviewers. We strongly encourage code deposition in a community repository (e.g. GitHub). See the Nature Research guidelines for submitting code & software for further information.

Data

Policy information about availability of data

All manuscripts must include a data availability statement. This statement should provide the following information, where applicable:

- Accession codes, unique identifiers, or web links for publicly available datasets
- A list of figures that have associated raw data
- A description of any restrictions on data availability

All data from this study are available from the corresponding author Z.Gao upon reasonable request. Please see data for all figures in the Resource Data file.

Field-specific reporting

Please select the one below that is the best fit for your research. If you are not sure, read the appropriate sections before making your selection.

☒ Life sciences ☐ Behavioural & social sciences ☐ Ecological, evolutionary & environmental sciences

For a reference copy of the document with all sections, see nature.com/documents/nr-reporting-summary-flat.pdf

Life sciences study design

All studies must disclose on these points even when the disclosure is negative.

Sample size	The sample sizes for electriphysiological and anatomical experiments were decided similar to the sample sizes used in this fields: more than 3 animals were used in each experiment. Power analysis was performed prior to all behavioral experiments and the sample sizes were assessed to meet the statistical requirements.<br>Please see references as:<br>(1) Nat Neurosci. 2015 Dec;18(12):1798-803. doi: 10.1038/nn.4167. Epub 2015 Nov 9.<br>(2) Nat Neurosci. 2018 May;21(5):725-735. doi: 10.1038/s41593-018-0129-x. Epub 2018 Apr 16.<br>(3) Nat Neurosci. 2017 May;20(5):727-734. doi: 10.1038/nn.4531. Epub 2017 Mar 20.
Data exclusions	No data was excluded.
Replication	All results were replicated at least in multiple animals (n > 3 mice). We gave at least 50 trials to the mice for each recording session. At least 5 mice were used for each physiological recording experiments. Behaviorial and anatomical experiments were replicated in at least 3 mice.
Randomization	Animals were randomized into experimental groups and control groups. In behavioral studies with optogenetic perturbation, perturbation trials were randomly allocated throughout the experiments.
Blinding	For all behavioral tests, animals in both the control and perturbation groups underwent the same condition, and most experiments were self-controlled (opto vs no-opto; pre-lesion vs post-lesion; pre-muscimol vs post-muscimol). Investigators were not blind during the experiments and data analysis.

Reporting for specific materials, systems and methods

We require information from authors about some types of materials, experimental systems and methods used in many studies. Here, indicate whether each material, system or method listed is relevant to your study. If you are not sure if a list item applies to your research, read the appropriate section before selecting a response.

Materials & experimental systems

n/a	Involved in the study	
☐	☒	Antibodies
☒	☐	Eukaryotic cell lines
☒	☐	Palaeontology and archaeology
☐	☒	Animals and other organisms
☒	☐	Human research participants
☒	☐	Clinical data
☒	☐	Dual use research of concern

Methods

n/a	Involved in the study	
☒	☐	ChIP-seq
☒	☐	Flow cytometry
☒	☐	MRI-based neuroimaging

Antibodies

Antibodies used	Goat ati-cholera toxin B subunit primary antibody (1:15000, List labs, 703)<br>Guinea pig anti-VGluT2 primary antibody (1:750, Sigma-Aldrich, AB2251-I)<br>Rabbit anti-Gad65/67 primary antibody (1:1000, Sigma-Aldrich, AB1511)<br>Goat anti-GFP primary antibody (1:5000, Rockland, 600-101-215)<br>Rabbit anti-NeuN primary antibody (1:1000, Millipore, abn78)<br>Biotinylated horse anti-goat secondary antibody (1:2000, Vector, BA-9500)<br>Alexa fluor® 488 donkey anti-guinea pig secondary antibody (1:400, Jackson, 706-545-148)<br>Alexa fluor® 488 donkey anti-rabbit secondary antibody (1:400, Jackson, 711-545-152)<br>Alexa fluor® 488 donkey anti-goat secondary antibody (1:200, Jackson, 705-545-147)<br>Cy™3 donkey anti-rabbit secondary antibody (1:400, Jackson, 711-165-152)
Validation	Here are the references for all antibody validation on mouse brain slices:<br>Goat ati-cholera toxin B subunit primary antibody: Science. 2019 May 24;364(6442). pii: eaaw0445. doi: 10.1126/science.aaw0445.<br>Guinea pig anti-VGluT2 primary antibody: Neuron. 2018 Apr 18;98(2):306-319.e7. doi: 10.1016/j.neuron.2018.03.010. Epub 2018 Apr

5.
Rabbit anti-Gad65/67 primary antibody: Nat Commun. 2018 Apr 12;9(1):1422. doi: 10.1038/s41467-018-03802-y.
Goat anti-GFP primary antibody: Neuron. 2017 Apr 19;94(2):337-346.e6. doi: 10.1016/j.neuron.2017.03.034.
Rabbit anti-NeuN primary antibody: Nature Communications. 2020 Apr 7;11(1):1729. doi: 10.1038/s41467-020-15537-w.
Biotinylated horse anti-goat secondary antibody: Nat Commun. 2019 Apr 15;10(1):1760. doi: 10.1038/s41467-019-09748-z.
Alexa fluor® 488 donkey anti-guinea pig secondary antibody: Nat Commun. 2020 Mar 13;11(1):1397. doi: 10.1038/s41467-020-15230-y.
Alexa fluor® 488 donkey anti-rabbit secondary antibody: Cell Reports. 2019 Jul 16;28(3):759-772.e10. doi: 10.1016/j.celrep.2019.06.058.
Alexa fluor® 488 donkey anti-goat secondary antibody: Nat Microbiol. 2017 Dec;2(12):1586-1591. doi: 10.1038/s41564-017-0057-7. Epub 2017 Nov 6.
Cy™3 donkey anti-rabbit secondary antibody: Nat Commun. 2020 Mar 13;11(1):1397. doi: 10.1038/s41467-020-15230-y.

Animals and other organisms

Policy information about studies involving animals; ARRIVE guidelines recommended for reporting animal research

Laboratory animals	We used both male and female mice aged 6-14 weeks in this study. The wild-type C57BL/6J (No. 000664) and transgenic Gad2-ires-Cre (No. 010802), VGluT2-ires-Cre (No. 016963), L7Cre (No. 004146) and Ai27D (No. 012567) mice were obtained from the Jackson Labs.
Wild animals	This study did not use wild animals.
Field-collected samples	This study did not use samples collected from the field.
Ethics oversight	All animal experiments in this study were approved by the institutional animal welfare committee of the Erasmus MC in accordance with Central Authority for Scientific Procedures on Animals guidelines issued by the Dutch Central Committee on Animal Experiments (CCD).

Note that full information on the approval of the study protocol must also be provided in the manuscript.
